# Supplementary material for: Impact of Invasive Pulmonary Aspergillosis in Critically Ill Surgical Patients with or without Solid Organ Transplantation
Source: J Clin Med. 2023 May 4;12(9):3282. doi: 10.3390/jcm12093282 (PMC10179688; doi:10.3390/jcm12093282)
Supplement: Supplementary file 1 [file jcm-12-03282-s001.zip › jcm-2276229-supplementary.pdf]

Supplementary Materials

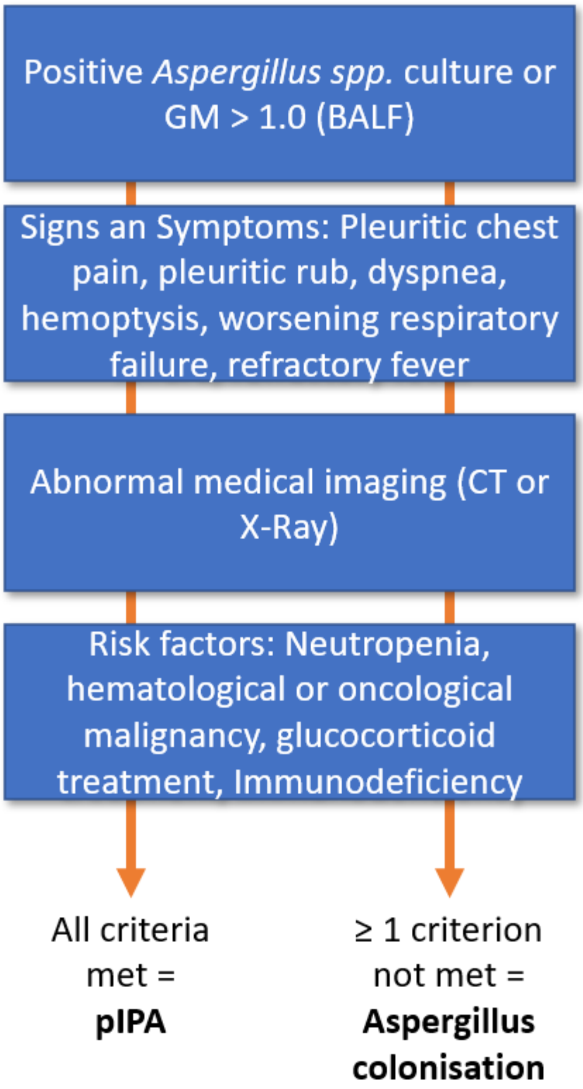

Figure S1. Diagnostic criteria of invasive pulmonary aspergillosis by Blot (1, 2) Abbreviations: *GM (Galactomannan); BALF* (Broncho-alveolar lavage fluid*), CT* (computed tomography), *pIPA* (putative invasive pulmonary aspergillosis).

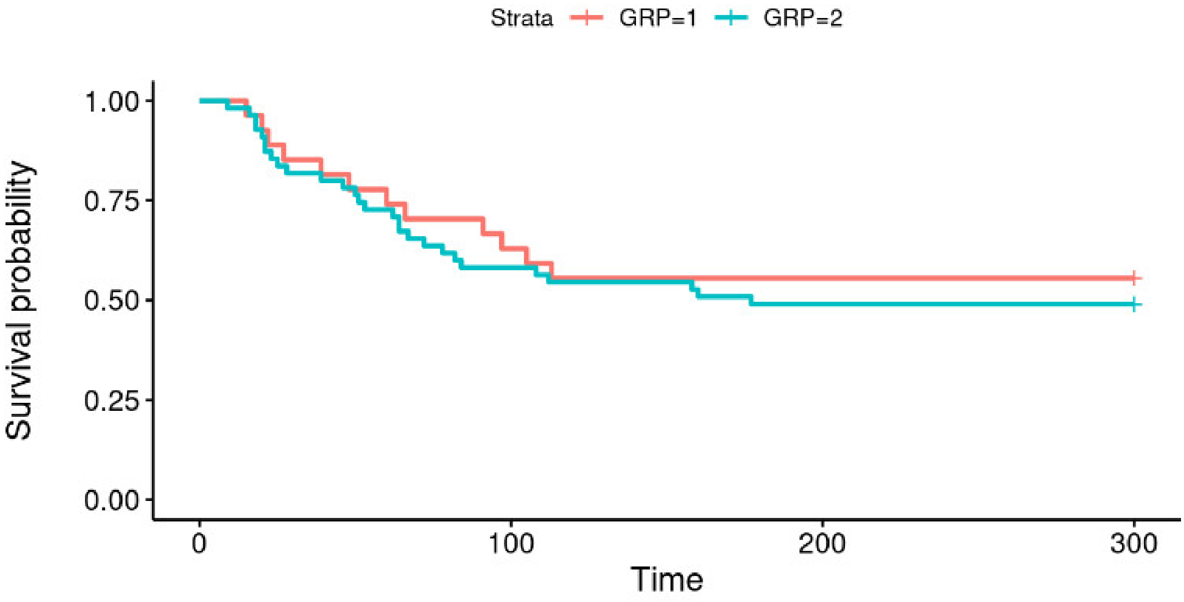

Figure S2. Kaplan-Meier curves of pIPA (GRP = 1)- versus non-pIPA patients (GRP = 2) in all patients included. Abbreviations: *IPA* (invasive pulmonary aspergillosis), *GRP* (Group).

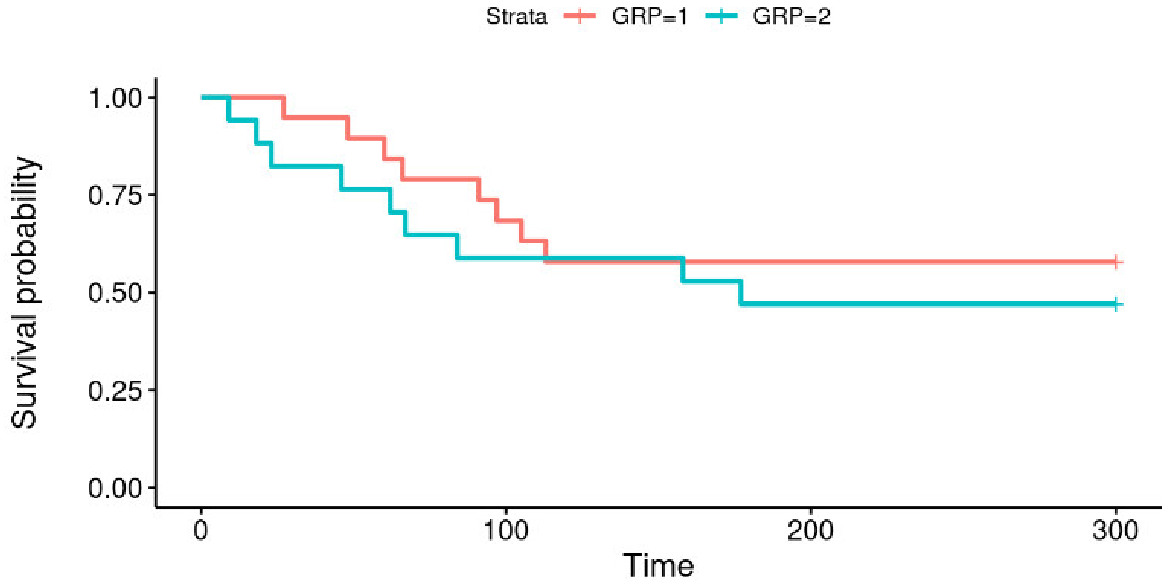

Figure S3. Kaplan-Meier curves of pIPA (GRP = 1)- versus non-pIPA patients (GRP = 2) in SOT patients only. Abbreviations: *IPA* (invasive pulmonary aspergillosis), *GRP* (Group), *SOT* (Solid organ transplantation).

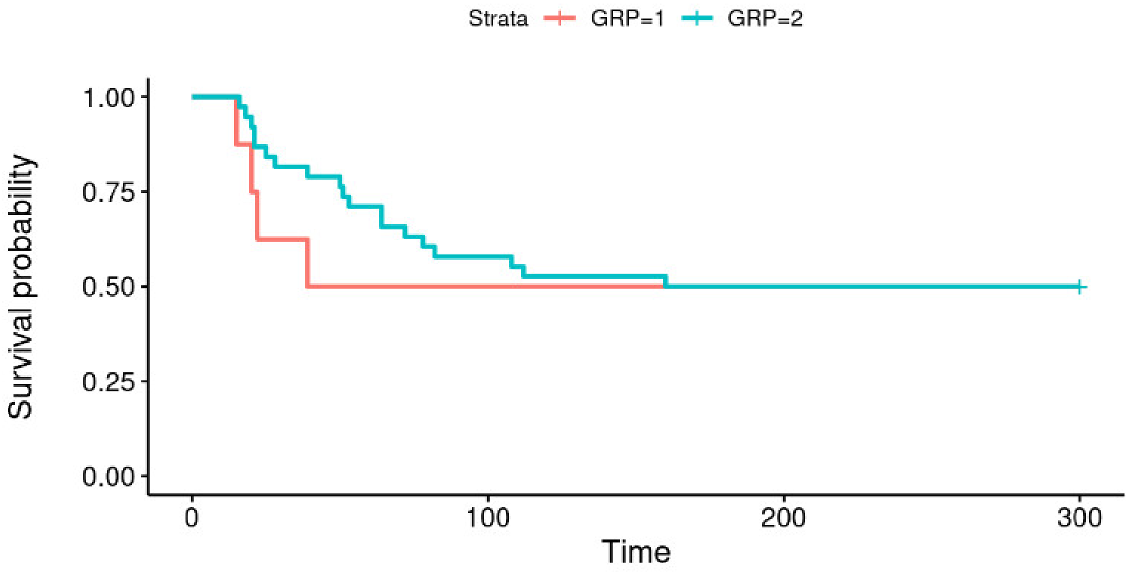

Figure S4. Kaplan-Meier curves of pIPA (GRP = 1)- versus non-pIPA patients (GRP = 2) in non-SOT patients only. Abbreviations: *IPA* (invasive pulmonary aspergillosis), *GRP* (Group), *SOT* (Solid organ transplantation).

Table S1. Diagnostic criteria of the AspICU clinical algorithm according to Blot et al. (2012)(1).

**Putative invasive pulmonary aspergillosis (all four criteria must be met)**
1. Aspergillus-positive lower respiratory tract specimen culture (entry criterion)
2. Compatible signs and symptoms (one of the following)
▪ Fever refractory to at least 3 d of appropriate antibiotic therapy
▪ Recrudescent fever after a period of effervescence of at least 48 h while still on antibiotics and without other apparent cause
▪ Pleuritic chest pain
▪ Pleuritic rub
▪ Dyspnea
▪ Hemoptysis
▪ Worsening respiratory insufficiency in spite of appropriate antibiotic therapy and ventilatory support
3. Abnormal medical imaging by portable chest X-ray or CT scan of the lungs
4. Either 4a or 4b
4a. Host risk factors (one of the following conditions)
▪ Neutropenia (absolute neutrophil count ,500/mm3) preceding or at the time of ICU admission
▪ Underlying hematological or oncological malignancy treated with cytotoxic agents
▪ Glucocorticoid treatment (prednisone equivalent, .20 mg/d)
▪ Congenital or acquired immunodeficiency
4b. Semiquantitative *Aspergillus*-positive culture of BAL fluid (1 or 11), without bacterial growth together with a positive cytological smear showing branching hyphae
Aspergillus **respiratory tract colonization**
When >1 criterion necessary for a diagnosis of putative IPA is not met, the case is classified as *Aspergillus* colonization.

Abbreviations: *BAL* (Bronchoalveolar lavage), *CT* (Computed tomography), *EORTC/MSG* (European Organization for the Research and Treatment of Cancer/Mycosis Study Group), *ICU* (Intensive care unit).

Table S1a. Microbiological tests and antifungal therapies of non-SOT patients.

	pIPA (*n* = 11)	Non-pIPA (*n* = 68)	*p*
SOFA first GM positivity †	15 (12; 15)	10 (8; 13)	0.010 *
BALF-positive GM			
First value of GM (BALF) †	2.63 (1.76; 6.05)	1.18 (0.71; 4.03)	0.012 *
Highest value of GM (BALF) †	2.63 (1.99; 6.16)	1.20 (0.71; 4.62)	0.009 **
Aspergillus spp. culture positive	5 (45)	17 (25)	0.3
Antifungal therapy‡			
Antifungal prophylaxis	0 (0)	5 (7.4)	0.9
Firstline therapy [yes]	8 (73)	38 (56)	0.3
Duration first line therapy [d] †	6 (2; 16)	4 (0; 12)	0.4
Time to first line therapy [d] †	8 (6; 12)	12 (4; 21)	0.6
Secondline therapy [yes]	3 (27)	9 (13)	0.4
Duration second line therapy [d] †	0 (0; 4)	0 (0; 0)	0.3
Rescue therapy [yes]	1 (9.1)	3 (4.4)	0.5

Abbreviations: *SOFA* (Sequential organ failure assessment), *ICU* (Intensive care unit), *GM* (Galactomannan), ODI (Optical Density Index), *BALF* (Broncho-alveolar lavage). Data are presented as *n* (%). † Values are presented as median, (Interquartile range). ‡ Antifungal therapy in in pIPA patients (Firstline therapy: 6x Voriconazole, 1x liposomal Amphotericin-B, 1x Caspofungin ;Secondline therapy: 1x Voriconazole, 1x Caspofungin, 1x inhalative Amphotericin-B; Rescue therapy: 1x Isavuconazole + liposomal Amphotericin-B. * $p < 0.05$, ** $p < 0.01$.

Table S1b. Microbiological tests and antifungal therapies of SOT patients.

	pIPA (*n* = 19)	Non-pIPA (*n* = 23)	*p*
SOFA first GM positivity †	13 (10; 14)	12 (8; 13)	0.3
BALF-positive GM			
First value of GM (BALF) †	4.60 (3.51; 5.70)	1.66 (0.74; 4.30)	0.005 **
Highest value of GM (BALF) †	6.36 (5.52; 7.60)	1.66 (0.74; 4.32)	<0.001 ***
Aspergillus spp. culture positive	9 (47)	4 (17)	0.036
Antifungal therapy ‡			
Antifungal prophylaxis	7 (37)	13 (57)	0.2
Firstline therapy [yes]	19 (100)	17 (74)	0.024 *
Duration first line therapy [d] †	24 (10; 45)	9 (2; 28)	0.031 *
Time to first line therapy [d] †	6 (4; 16)	13 (9; 31)	0.042 *
Secondline therapy [yes]	12 (63)	5 (22)	0.006 *
Duration second line therapy [d] †	11 (0; 18)	0 (0; 0)	0.006 *
Rescue therapy [yes]	5 (26)	1 (4.3)	0.075

Abbreviations: *SOFA* (Sequential organ failure assessment), *ICU* (Intensive care unit), *GM* (Galactomannan), ODI (Optical Density Index), *BALF* (Broncho-alveolar lavage). Data are presented as *n* (%). ‡ Antifungal therapy in in pIPA patients (Firstline therapy: 10x Voriconazole, 7x Amphotericin-B, 1x Isavuconazole, 1x inhalative Amphotericin-B; Secondline therapy: 4x Voriconazole, 3x liposomal Amphotericin-B, 2x Caspofungin, 3x Isavuconazole; Rescue therapy: 1x liposomal Amphotericin-B, 1x Voriconazole + Posaconazole + increase in dosage of liposomal Amphotericin-B, 1x Micafungin, 1x Posaconazole, 1x a. Caspofungin + Isovuconazole + Ampho-B (inh.) b. Liposomal Amphotericin-B (IV) + Anidulafungin + Amphotericin-B (inh.), c) b. + Voriconazole. † Values are presented as median, (Interquartile range). * $p < 0.05$, ** $p < 0.01$, *** $p < 0.001$.

Table S2. Antifungal therapy in patients with pIPA.

	**Firstline †** **(*n* = 27)**	**Secondline †** **(*n* = 15)**	**Rescue** **(*n* = 6)**
Voriconazole	16	5	
Liposomal Amphotericin-B (IV)	8	3	1
Caspofungin	1	3	
Isavuconazole	1	3	
a. Caspofungin + Isovuconazole + Ampho-B (inh.) b. Liposomal Amphotericin-B (IV) + Anidulafungin + Amphotericin-B (inh.) c. b + Voriconazole			1
Isavuconazole + Liposomal Amphotericin-B (IV)			1
Posaconazole			1
Micafungin			1
Voriconazol + Posaconazol + increase in dosage of Liposomal Amphotericin-B (IV)			1

Abbreviations: *IV* (Intravenous), *inh* (inhalation). † 1 patient in firstline therapy group and 1 patient in secondline therapy group with liposomal Amphotericin-B inhalation therapy.

Table S3a. Risk factors for death within 28 days after ICU admission in non-SOT patients.

	**Hazard ratio (95% CI)**	***p***
**Univariate Analysis**		
Bacteremia	3.5 (1.6–7.9)	0.002**
Candidemia	0.88 (0.12–6.5)	0.9
SOFA ICU admission	1.1 (0.99–1.2)	0.074
GM-SOFA first GM positivity	1.3 (1.1–1.5)	<0.001***
First value of GM (BALF)	1.2 (1–1.4)	0.047*
Highest value of GM (BALF)	1.1 (0.96–1.3)	0.15
Putative IPA	0.41 (0.16–1)	0.057
Firstline therapy	0.51 (0.21–1.2)	0.13
Time to first line therapy	1 (0.98–1)	0.96
Secondline therapy	0.39 (0.16–0.95)	0.037
Rescue therapy	0.64 (0.15–2.7)	0.55
Mechanical ventilation	1 (1–1)	0.22
**Multivariate Analysis**		
Bacteremia	2.38 (0.95–5.98)	0.065
Mechanical ventilation	1.00 (1.00 – 1.00)	0.028
GM-SOFA first GM positivity	1.33 (1.14–1.56)	<0.001***
Putative IPA	1.02 (0.33–3.12)	0.980

Abbreviations: *SOFA* (Sequential organ failure assessment), *ICU* (Intensive care unit), *GM* (Galactomannan), *BALF* (Broncho-alveolar lavage), *IPA* (invasive pulmonary aspergillosis). * $p < 0.05$, ** $p < 0.01$, *** $p < 0.001$.

Table S3b. Risk factors for death within 28 days after ICU admission in SOT patients.

	Hazard ratio (95% CI)	p
**Univariate Analysis**		
Bacteremia	5.9 (1.4–25)	0.015*
Candidemia	9.3 (1–83)	0.046*
SOFA ICU admission	0.87 (0.74–1)	0.093
GM-SOFA first GM positivity	1.2 (0.95–1.4)	0.15
First value of GM (BALF)	1.1 (0.81–1.4)	0.62
Highest value of GM (BALF)	1 (0.81–1.3)	0.8
Putative IPA	1.3 (0.32–5.6)	0.69
Firstline therapy	4.1e–09 (0-Inf)	1
Time to first line therapy	1 (1–1)	0.1
Secondline therapy	0.58 (0.14–2.3)	0.44
Rescue therapy	1.1 (0.13–8.9)	0.94
Mechanical ventilation	1 (1–1)	0.95
**Multivariate Analysis**		
Bacteremia	6.14 (1.31–28.73)	0.021*
Mechanical ventilation	1.00 (1.00- 1.00)	0.916
GM-SOFA first GM positivity	1.14 (0.93–1.40)	0.206
Putative IPA	1.53 (0.36–6.53)	0.563

Abbreviations: *SOFA* (Sequential organ failure assessment), *ICU* (Intensive care unit), *GM* (Galactomannan), *BALF* (Broncho-alveolar lavage), *IPA* (invasive pulmonary aspergillosis). * $p < 0.05$.

Table S4a. Risk factors for an unfavourable outcome within 28 days after ICU admission in the whole cohort patients.

	Hazard ratio (95% CI)	p
**Univariate analysis**		
Bacteremia	2.3 (1.5–3.7)	<0.001***
Candidemia	1.8 (0.64–4.8)	0.27
SOFA ICU admission	1 (1–1.1)	0.047
GM-SOFA first GM positivity	1.1 (1.1–1.2)	<0.001***
First value of GM (BALF)	1.1 (0.98–1.2)	0.12
Highest value of GM (BALF)	1.1 (1–1.2)	0.021
Putative IPA	0.66 (0.42–1)	0.073
Firstline therapy	0.42 (0.26–0.68)	<0.001***
Time to first line therapy	1 (0.99–1)	0.28
Secondline therapy	0.42 (0.27–0.66)	<0.001***
Rescue therapy	0.43 (0.22–0.84)	0.013*
Mechanical ventilation	1 (1–1)	<0.001
**Multivariate Analysis**		
Bacteremia	2.18 (1.33–3.59)	0.002**
Mechanical ventilation	1.00 (1.00 – 1.00)	0.001
GM-SOFA first GM positivity	1.12 (1.06–1.19)	<0.001***
Putative IPA	1.08 (0.65–1.78)	0.777

Abbreviations: *SOFA* (Sequential organ failure assessment), *ICU* (Intensive care unit), *GM* (Galactomannan), *BALF* (Broncho-alveolar lavage), *IPA* (invasive pulmonary aspergillosis). * $p < 0.05$, ** $p < 0.01$, *** $p < 0.001$.

Table S4b. Risk factors for an unfavourable outcome within 28 days after ICU admission in non-SOT patients.

	Hazard ratio (95% CI)	*p*
**Univariate analysis**		
Bacteremia	3.2 (1.8–6)	<0.001***
Candidemia	1.6 (0.5–5.1)	0.43
SOFA ICU admission	1.1 (1–1.1)	0.056
GM-SOFA first GM positivity	1.2 (1.1–1.3)	<0.001***
First value of GM (BALF)	1.1 (0.99–1.3)	0.08
Highest value of GM (BALF)	1.1 (0.99–1.3)	0.063
Putative IPA	0.54 (0.26–1.1)	0.092
Firstline therapy	0.45 (0.25–0.79)	0.005*
Time to first line therapy	1 (0.99–1)	0.54
Secondline therapy	0.3 (0.16–0.59)	<0.001***
Rescue therapy	0.4 (0.14–1.1)	0.083
Mechanical ventilation	1 (1–1)	0.002
**Multivariate Analysis**		
Bacteremia	2.70 (1.41–5.16)	0.002**
Mechanical ventilation	1.00 (1.00–1.00)	0.018
GM-SOFA first GM positivity	1.18 (1.08–1.29)	<0.001***
Putative IPA	1.11 (0.48–2.54)	0.81

Abbreviations: *SOFA* (Sequential organ failure assessment), *ICU* (Intensive care unit), *GM* (Galactomannan), *BALF* (Broncho-alveolar lavage), *IPA* (invasive pulmonary aspergillosis). * $p < 0.05$, ** $p < 0.01$, *** $p < 0.001$.

Table S4c. Risk factors for an unfavourable outcome within 28 days after ICU admission in SOT patients.

	Hazard ratio (95% CI)	*p*
**Univariate analysis**		
Bacteremia	1.3 (0.61–2.6)	0.52
Candidemia	5.3 (0.66–43)	0.12
SOFA ICU admission	1 (0.95–1.1)	0.54
GM-SOFA first GM positivity	1.1 (0.99–1.2)	0.074
First value of GM (BALF)	0.99 (0.88–1.1)	0.91
Highest value of GM (BALF)	1.1 (0.94–1.2)	0.34
Putative IPA	0.89 (0.46–1.7)	0.73
Firstline therapy	0.4 (0.14–1.1)	0.09
Time to first line therapy	1 (0.99–1)	0.39
Secondline therapy	0.62 (0.32–1.2)	0.16
Rescue therapy	0.49 (0.2–1.2)	0.12
Mechanical ventilation	1 (1–1)	0.008
**Multivariate Analysis**		
Bacteremia	1.49 (0.68–3.28)	0.320
Mechanical ventilation	1.00 (1.00–1.00)	0.021
GM-SOFA first GM positivity	1.06 (0.96–1.16)	0.244
Putative IPA	1.024 (0.50–2.10)	0.949

Abbreviations: *SOFA* (Sequential organ failure assessment), *ICU* (Intensive care unit), *GM* (Galactomannan), *BALF* (Broncho-alveolar lavage), *IPA* (invasive pulmonary aspergillosis).

Table S5a. Risk factors for death within 90 days after ICU admission in the whole cohort of patients.

	Hazard ratio (95% CI)	*p*
**Univariate Analysis**		
Bacteremia	3 (1.6–5.8)	<0.001***
Candidemia	1.5 (0.37–6.4)	0.56
SOFA ICU admission	1 (0.97–1.1)	0.28
GM-SOFA first GM positivity	1.2 (1.1–1.3)	<0.001***
First value of GM (BALF)	1.1 (0.94–1.2)	0.33
Highest value of GM (BALF)	1.1 (0.93–1.2)	0.4
Putative IPA	0.89 (0.43–1.8)	0.75
Firstline therapy	0.43 (0.19–0.98)	0.044*
Time to first line therapy	0.99 (0.97–1)	0.48
Secondline therapy	0.49 (0.25–0.95)	0.035*
Rescue therapy	0.61 (0.24–1.6)	0.3
Mechanical ventilation	1 (1–1)	0.64
**Multivariate Analysis**		
Bacteremia	2.56 (1.27–5.17)	0.009**
Mechanical ventilation	1.00 (1.00 – 1.00)	0.485
GM-SOFA first GM positivity	1.25 (1.12–1.39)	<0.001***
Putative IPA	1.48 (0.67–3.24)	0.329

Abbreviations: *SOFA* (Sequential organ failure assessment), *ICU* (Intensive care unit), *GM* (Galactomannan), *BALF* (Broncho-alveolar lavage), *IPA* (invasive pulmonary aspergillosis), * $p < 0.05$, ** $p < 0.01$, *** $p < 0.001$.

Table S5b. Risk factors for death within 90 days after ICU admission in non-SOT patients.

	Hazard ratio (95% CI)	*p*
**Univariate Analysis**		
Bacteremia	3.9 (1.8–8.5)	<0.001***
Candidemia	0.84 (0.11–6.2)	0.87
SOFA ICU admission	1.1 (1–1.2)	0.041
GM-SOFA first GM positivity	1.3 (1.2–1.5)	<0.001***
First value of GM (BALF)	1.2 (0.99–1.4)	0.066
Highest value of GM (BALF)	1.1 (0.95–1.3)	0.19
Putative IPA	0.43 (0.17–1.1)	0.067
Firstline therapy	0.43 (0.18–1)	0.055
Time to first line therapy	0.99 (0.97–1)	0.55
Secondline therapy	0.29 (0.13–0.66)	0.003**
Rescue therapy	0.43 (0.13–1.4)	0.17
Mechanical ventilation	1 (1–1)	0.81
**Multivariate Analysis**		
Bacteremia	2.69 (1.14–6.32)	0.023*
Mechanical ventilation	1.00 (1.00–1.00)	0.535
GM-SOFA first GM positivity	1.32 (1.14–1.53)	<0.001***
Putative IPA	1.08 (0.37–3.16)	0.886

Abbreviations: *SOFA* (Sequential organ failure assessment), *ICU* (Intensive care unit), *GM* (Galactomannan), *BALF* (Broncho-alveolar lavage), *IPA* (invasive pulmonary aspergillosis). * $p < 0.05$, ** $p < 0.01$, *** $p < 0.001$.

Table S5c. Risk factors for death within 90 days after ICU admission in SOT patients.

	Hazard ratio (95% CI)	*p*
**Univariate Analysis**		
Bacteremia	1.9 (0.55–6.5)	0.31
Candidemia	5.3 (0.66–43)	0.12
SOFA ICU admission	0.99 (0.87–1.1)	0.87
GM-SOFA first GM positivity	1.2 (0.98–1.4)	0.086
First value of GM (BALF)	0.96 (0.76–1.2)	0.76
Highest value of GM (BALF)	1 (0.85–1.3)	0.67
Putative IPA	1.5 (0.45–5.3)	0.49
Firstline therapy	4.2e–09 (0-Inf)	1
Time to first line therapy	1 (0.97–1)	0.86
Secondline therapy	0.76 (0.23–2.5)	0.66
Rescue therapy	0.74 (0.16–3.4)	0.7
Mechanical ventilation	1 (1–1)	0.57
**Multivariate Analysis**		
Bacteremia	2.03 (0.55–7.48)	0.289
Mechanical ventilation	1.00 (1.00–1.00)	0.990
GM-SOFA first GM positivity	1.16 (0.96–1.39)	0.117
Putative IPA	1.55 (0.45–5.40)	0.491

Abbreviations: *SOFA* (Sequential organ failure assessment), *ICU* (Intensive care unit), *GM* (Galactomannan), *BALF* (Broncho-alveolar lavage), *IPA* (invasive pulmonary aspergillosis).

Table S6a. Risk factors for an unfavourable outcome within 90 days after ICU admission in the whole cohort of patients.

	Hazard ratio (95% CI)	*p*
**Univariate Analysis**		
Bacteremia	2.2 (1.3–3.9)	0.005**
Candidemia	1 (0.25–4.3)	0.96
SOFA ICU admission	1 (0.98–1.1)	0.16
GM-SOFA first GM positivity	1.2 (1.1–1.3)	<0.001***
First value of GM (BALF)	1.1 (1–1.3)	0.01*
Highest value of GM (BALF)	1.1 (1–1.2)	0.01*
Putative IPA	0.74 (0.42–1.3)	0.3
Firstline therapy	0.33 (0.17–0.66)	0.002*
Time to first line therapy	1 (0.99–1)	0.31
Secondline therapy	0.37 (0.22–0.62)	<0.001***
Rescue therapy	0.33 (0.17–0.67)	0.002**
Mechanical ventilation	1 (1–1)	<0.001
**Multivariate Analysis**		
Bacteremia	2.22 (1.21–4.08)	0.01*
Mechanical ventilation	1.00 (1.00–1.00)	0.01
GM-SOFA first GM positivity	1.12 (1.04–1.21)	0.002**
Putative IPA	1.31 (0.68–2.53)	0.42

Abbreviations: *SOFA* (Sequential organ failure assessment), *ICU* (Intensive care unit), *GM* (Galactomannan), *BALF* (Broncho-alveolar lavage), *IPA* (invasive pulmonary aspergillosis). * $p < 0.05$, ** $p < 0.01$, *** $p < 0.001$.

Table S6b. Risk factors for an unfavourable outcome within 90 days after ICU admission in non-SOT patients.

	Hazard ratio (95% CI)	*p*
**Univariate Analysis**		
Bacteremia	3.4 (1.7–6.9)	<0.001***
Candidemia	0.67 (0.092–4.9)	0.69
SOFA ICU admission	1.1 (1–1.2)	0.025*
GM-SOFA first GM positivity	1.2 (1.1–1.4)	<0.001***
First value of GM (BALF)	1.2 (1.1–1.4)	0.007**
Highest value of GM (BALF)	1.2 (1–1.3)	0.044*
Putative IPA	0.38 (0.17–0.85)	0.018*
Firstline therapy	0.41 (0.19–0.89)	0.023*
Time to first line therapy	1 (0.98–1)	0.75
Secondline therapy	0.22 (0.11–0.45)	<0.001***
Rescue therapy	0.34 (0.12–0.96)	0.042*
Mechanical ventilation	1 (1–1)	0.016
**Multivariate Analysis**		
Bacteremia	2.72 (1.27–5.84)	0.010**
Mechanical ventilation	1.00 (1.00–1.00)	0.06*
GM-SOFA first GM positivity	1.21 (1.08–1.35)	0.001**
Putative IPA	0.87 (0.34–2.22)	0.776

Abbreviations: *SOFA* (Sequential organ failure assessment), *ICU* (Intensive care unit), *GM* (Galactomannan), *BALF* (Broncho-alveolar lavage), *IPA* (invasive pulmonary aspergillosis). * $p < 0.05$, ** $p < 0.01$, *** $p < 0.001$.

Table S6c. Risk factors for unfavourable outcome within 90 days after ICU admission in SOT patients.

	Hazard ratio (95% CI)	*p*
**Univariate Analysis**		
Bacteremia	1 (0.41–2.6)	0.96
Candidemia	5.3 (0.66–43)	0.12
SOFA ICU admission	0.97 (0.89–1.1)	0.55
GM-SOFA first GM positivity	1.1 (0.97–1.2)	0.16
First value of GM (BALF)	1 (0.89–1.2)	0.61
Highest value of GM (BALF)	1.1 (0.95–1.3)	0.19
Putative IPA	1.6 (0.7–3.6)	0.27
Firstline therapy	0.15 (0.021–1.1)	0.068
Time to first line therapy	1 (0.99–1)	0.24
Secondline therapy	0.71 (0.32–1.6)	0.4
Rescue therapy	0.34 (0.13–0.88)	0.026 *
Mechanical ventilation	1 (1–1)	0.0035
**Multivariate Analysis**		
Bacteremia	1.269 (0.4523–3.561)	0.6507
Mechanical ventilation	1.001 (1.0001–1.001)	0.0163
GM-SOFA first GM positivity	1.028 (0.9229–1.145)	0.6158
Putative IPA	2.038 (0.7815–5.313)	0.1455

Abbreviations: *SOFA* (Sequential organ failure assessment), *ICU* (Intensive care unit), *GM* (Galactomannan), *BALF* (Broncho-alveolar lavage), *IPA* (invasive pulmonary aspergillosis). * $p < 0.05$.

Table S7. Suspected infectious foci in all pIPA- and non-pIPA patients with bacteremia.

	**pIPA (n = 10)**	**Non-pIPA (n = 17)**
Suspected source of infection		
Pulmonary	1	6
Abdominal	5	7
Catheter associated		1
Contamination/Unknown	4	3

References

1. Blot, S.I.; Taccone, F.S.; Van den Abeele, A.-M.; Bulpa, P.; Meersseman, W.; Brusselaers, N.; Dimopoulos, G.; Paiva, J.A.; Misset, B.; Rello, J.; et al. A Clinical Algorithm to Diagnose Invasive Pulmonary Aspergillosis in Critically Ill Patients. *Am. J. Respir. Crit. Care Med.* **2012**, *186*, 56–64.
2. Ledoux, M.-P.; Herbrecht, R. Invasive Pulmonary Aspergillosis. *J. Fungi* **2023**, *9*, 131. https://doi.org/10.3390/jof9020131
